# Dr. Sajous’ Great Work*The Internal Secretions and The Principles of Medicine, F. A. Davis Company, Philadelphia, Vol. II, Biology, Pharmacodynamics, Pathogenesis, Applied Therapeutics.

**Published:** 1907-12

**Authors:** 


### DR. SAJOUS' GREAT WORK.*

Dr. Sajous has made a wonderful contribution to medical science, and his name will be handed down to all future generations, along with that of Pasteur, Metchnikoff, Virchow, Koch, Wright, Foster and Lister. He has solved the problem of glandular action and pointed out the rôle that the secretions of the ductless glands perform, not only in nutrition and metabolism, but in resisting disease and overcoming pathogenic invasion. He, like

---

*The Internal Secretions and The Principles of Medicine, F. A. Davis Company, Philadelphia, Vol. II, Biology, Pharmacodynamics, Pathogenesis, Applied Therapeutics.

another Œdipus, has solved the riddle of the Sphynx;—the thyroid gland is the source of that solvent principle in the blood—opsonins—of which so much is heard these days, and which Metchnikoff says is a product of the phagocytes themselves—the chief defenders of the system against disease.

In this issue I reproduce Dr. J. M. Taylor's splendid article, "The Future Science of Medicine,"† and ask my readers to read it. It is intensely interesting and instructive. It is a revelation and a better "review" than I can write. It gives a succinct summary of prominent features of Dr. Sajous' discoveries: the cardinal rôle of the ductless glands in the defense of the body against disease, and (what is of still greater importance to the practicing physician) the fact that these defensive functions can be enhanced by appropriate remedies. This affords, obviously, a new and scientific foundation for therapeutics, a branch of medicine which has lost in great part, in recent years, the confidence of the profession. The righ rank that Dr. Sajous' labors have recently been given in America and on the continent of Europe and the fact that they elucidate a very large number of admittedly obscure questions, make his work not only timely but of the greatest interest and importance.

